# Case Control Analyses of Acute Endophthalmitis after Cataract Surgery in South India Associated with Technique, Patient Care, and Socioeconomic Status

**DOI:** 10.1155/2012/298459

**Published:** 2012-02-23

**Authors:** Taraprasad Das, Anjli Hussain, Thomas Naduvilath, Savitri Sharma, Subhadra Jalali, Ajit B. Majji

**Affiliations:** ^1^Smt. Kanuri Santhamma Retina Vitreous Center, L V Prasad Eye Institute, Hyderabad 500034, India; ^2^L V Prasad Eye Institute, Patia, Bhubaneswar 751024, India; ^3^Public Health Ophthalmology Division, L V Prasad Eye Institute, Hyderabad 500034, India; ^4^Brien Holden Vision Institute, Sydney, NSW 2052, Australia; ^5^Javeri Microbiology Center, L V Prasad Eye Institute, Hyderabad 500034, India

## Abstract

*Purpose*. We investigated acute endophthalmitis incidence following cataract surgery vis-a-vis the current technological and postoperative care changes in higher and lower socioeconomic categories of patients in South India. *Methods*. In a retrospective case control study, we analyzed 62 cases of acute endophthalmitis and 5 controls for each endophthalmitis case from 46,095 cataract surgeries done between years 1993 and 1998. The time period covered the transition of surgical technique and after care. In addition, we analyzed systemic diseases, surgeon factor, habitat, and socioeconomic status. *Results*. Clinical and culture positive endophthalmitis incidence were 0.13% and 0.07%, respectively. Differential incidence of 0.10% and 0.17% for in- and ambulatory care surgeries, respectively, was close to statistical significance (*P* = 0.054). Lower economy category ambulatory patients had higher risk of infection. *Conclusion*. Ambulatory cataract surgery carried additional risk for post-operative infection in lower socioeconomic group. Improved health education could ensure greater safety.

## 1. Introduction

The current standard of cataract surgery is small incision cataract surgery and phacoemulsification. The postoperative care has changed from admission in the hospital for several days to total ambulatory care. European and North American studies have examined the safety and early recovery of patients and have justified this change [1–3]. The less developed countries including India have also adopted this change in technique and patient care. A nationwide or large single-center study on the safety and complications related to these changes is sparse in India. We believe these studies will help planning the management policies of cataract surgery and finally formulate a uniform health care planning in India.

There were three objectives of this study: (1) to estimate the rate of acute endophthalmitis in a large tertiary care eye hospital in South India, (2) to correlate the events of endophthalmitis with change in surgical technique from extracapsular cataract extraction (ECCE) to phacoemulsification and from the inpatient to ambulatory patient care, and (3) to evaluate the differential infection rate in the higher and lower socioeconomic strata of the society.

## 2. Patients and Methods

The study was done in a large tertiary care referral eye center in South India. Currently, the institute performs about 12,000 adult cataract surgeries annually. Comprehensive and total eye care is provided to patients of lower socioeconomic group (nonpaying patients) at no cost to the family. The materials for this study were obtained from the patient records of the institute after due clearance from the institutional review board. The research adhered to the tenets of the declaration of Helsinki.

All adult cataract surgeries (excluding 2114 complicated cataracts following uveitis and trauma) performed between January 1993 and December 1998 were included. The study did not include the referred patients of postcataract surgery acute endophthalmitis operated outside the institute facilities. All patients were operated either by institute full-time faculty or ophthalmology fellows after a sufficient period of training (certified by the faculty). Apart from specific technique related to the ECCE and phacoemulsification, all processes related to pre-, intra-, and postoperative care were uniform as per the institute protocol earlier published by us [4]. Irrespective of inpatient or ambulatory care, the operated eye was patched overnight. The inpatient care patients stayed overnight in the institute, and the ambulatory care patients were discharged usually within one hour of surgery.

The eye patch was removed in all patients on the first postoperative day and replaced with a plastic eye shield or a pair of protective goggles. The first day evaluation included uncorrected and pinhole Snellen acuity under standard conditions, applanation tonometry, a detailed slit lamp examination of the anterior segment, and fundus biomicroscopy using a +78/90 D lens. The postoperative medications included topical fluoroquinolones four times daily for two weeks and topical 1% prednisolone acetate six times daily for four weeks and tapered thereafter. Further followup schedule was at weeks 1, 4, and 12. All patients were routinely instructed to report immediately should they notice/experience increased redness, pain, unusual discharge from the eye, and reduction in vision.

All patients who returned with these symptoms and were suspected to have inflammatory or infective endophthalmitis were examined in the retina-vitreous service (TD, SJ, ABM) for further management as per the standards we have earlier published [4]. Prior to December 1995 (publication of EVS results [5]), all patients received immediate vitrectomy; beginning from January 1996, the EVS recommendations for postcataract surgery endophthalmitis were followed, though surgeon-specific variations were allowed. In the pretransition period (1993–1995), all patients also received intravenous antibiotics, and this was discontinued after publication of the EVS results.

The collected undiluted vitreous fluid was evaluated in the microbiology laboratory for microscopy and culture (aerobic and anaerobic bacteria and fungi) as per the institutional protocol [6]. All cultures were kept at least for a period of 4 days (14 days when fungus was suspected) before declaring them negative. A positive culture was defined as confluent growth of organism(s) at the site of inoculation on one solid medium and nonconfluent growth in one solid medium along with growth in one or more liquid media; growth of the same microorganism in one liquid medium which was also identified in microscopy. Patients were kept admitted to the institute for a period of 3 to 5 days (five days when patients received intravenous antibiotics). During this period, they were treated with intensive topical antibiotics, topical cycloplegic, intensive topical, and oral corticosteroids (except in cases of suspected or confirmed fungal endophthalmitis).

### 2.1. Case Control

Case control analysis was done for the purpose of identifying all factors presumably associated with acute clinical endophthalmitis. Five controls per case were selected from the surgical registry amongst all cataract surgeries done in the same time period. A control was defined as a cataract-operated subject without acute endophthalmitis. The control for each case was identified using the systematic random sampling strategy from the entire time period of the study. They were systematically chosen from the chronologically sorted list of cataract surgeries done in this period. This method of ascertaining the controls was adopted so that the effect of factors such as change in surgical technique and patient care was not lost. The analyzed factors included the type of patient care (inpatient and ambulatory), economic status (higher and lower), type of surgery (ECCE and phacoemulsification; IOL and no IOL implantation), systemic conditions (diabetes and hypertension), habitat (city limit and outstation), and surgeon factor (faculty and fellow-in-training).

The years 1993–1995 were termed *pretransition period, *and the years 1996–1998 were termed *posttransition period. *The transition was from the ECCE and inpatient postoperative care in 1993–1995 to phacoemulsification and ambulatory postoperative care in 1996–1998. The 1993–1995 period also served as EVS prepublication time, and the 1996–1998 served as EVS postpublication time.

### 2.2. Statistical Analysis

The incidence of acute endophthalmitis in the entire sample was compared between patient groups using Fisher's exact test. The risk of endophthalmitis was compared between patient groups using risk ratios and their 95% confidence intervals (CIs). Independent factors in the case-control study were tested for significance in univariate level using Fisher's exact test, *t*-tests, unadjusted odds ratios, and adjusted odds ratios using logistic regression in the multivariate level. Significant factors in the univariate analysis at *P* < 0.15 were used for further multivariate testing. Factors and interactions of factors were considered significant at *P* < 0.05. STATA-7 Intercooled STATA for Windows 7.0 (Texas, 2001) was used for all statistical analyses.

## 3. Results

In the study period 1993–1998, a total of 46,095 cases of uncomplicated adult cataract extraction with IOL implantation surgeries were performed—23,727 (51.48%) paying and 22,368 (48.52%) nonpaying patients. The transition time marked shift in technology of surgery and techniques of patient care and also simultaneously coincided with the publication of the first EVS report [5]. In pretransition period, 20,039 patients (paying: 10,560; nonpaying: 9,479) and in posttransition period, 26,056 (paying: 13,167; nonpaying: 12,889) were operated for cataract. In the former period, all patients received ECCE (with/without IOL) and were treated as inpatients. In the later period, 80% (20,848 of 26,056) of patients received phacoemulsification, and in 95% of instances (24,752 of 26,056), the patients were provided ambulatory care.

Based on the clinical examination, acute endophthalmitis was suspected in 62 patients, with an incidence of 0.13% (62 of 46,095). There were 38 (61.3%) males. Thirty six vitreous samples were culture positive, a rate of 58.06% (36 of 62), and 37 microorganisms were isolated (one sample had polymicrobial infection). Thus, the incidence of culture-proven endophthalmitis was 0.07%. The interval between cataract surgery and presentation with symptoms and signs of endophthalmitis was  15 ± 12  days. The patient's age ranged from 42 to 81 years (mean 52 + 11 years; median 60 years). Primary surgery was ECCE in 4 (6.5%) eyes, ECCE and IOL implantation in 42 (67.7%) eyes, and phacoemulsification and IOL implantation in 16 (25.8%) eyes. Primary pars plana vitrectomy was done in 41 (66.1%) eyes, and primary vitreous biopsy in the remaining 21 (33.9%) eyes. IOL was explanted in 6 (9.7%) eyes during primary vitrectomy. Three patients in the vitreous biopsy group (3 of 21; 14.3%) needed vitrectomy. Thus, 44 (71%) eyes needed vitrectomy. All patients received two intraocular antibiotics (cefazoline/vancomycin + amikacin/ceftazidime), and the antibiotics (culture adjusted) were repeated in 3 patients who had received deferred vitrectomy. Sixteen of 37 microorganisms (43.2%) were *Staphylococcus epidermidis, *and five (13.5%) were *Pseudomonas aeruginosa *(Table 1). Gram-positive cocci (GPC) grew in 64.9% instances (24 of 37 growths); Gram-negative bacilli (GNB) grew in 24.3% instances (9 of 37 growths). Eleven of 62 patients (17.7%) in the entire series and 8 of 36 (22.2%) culture-positive patients regained a final visual acuity of 20/40 or better. Thirty five of 62 (56.5%) patients in the entire series and 21 of 36 (58.3%) culture-proven infected eyes obtained final acuity of 20/100 or better.

The total and differential incidence rates of endophthalmitis are given in Table 2. The incidence of endophthalmitis was higher in the ambulatory patient care group compared to the inpatient care group, and this difference was statistically significant (*P* = 0.054). This difference in the incidence of endophthalmitis rates was not observed in the higher socioeconomic group of patients (*P* = 0.351).

### 3.1. Case Control Analysis

The age and gender distribution of cases and controls was not significant (Table 3; univariate analysis). Ambulatory patient care (*P* = 0.025) and patients residing within the city limit (*P* = 0.04) were significantly associated with the cases. The low socioeconomic group of patients was a significant factor at the 10% level (*P* = 0.157). The significance of patient residence in the multivariate analyses may be related to sampling variations of the case control itself.

Table 4 shows the results of multivariate analysis of factors associated with endophthalmitis. The interaction of socioeconomic status with type of patient care was a significant factor (*P* = 0.038) (Tables 5 and 6). The risk of infection was higher in ambulatory patient care of nonpaying patients (lower socioeconomic status) compared to inpatient care (*P* = 0.001) and compared to all paying patients (*P* = 0.001). All paying patients (higher socioeconomic patients) and all inpatient care were not associated with a higher risk of infection. Type of cataract surgery (ECE or phacoemulsification) systemic disease (diabetes and hypertension) and surgeon factor (faculty and fellow) did not have statistical significance in univariate analysis.

## 4. Discussion

The current tertiary eye care study center in South India caters to both higher and lower socioeconomic group without change in quality of care. The nonpaying category of patients are those who possess BPL (below poverty line) card issued by the state government. A single large center ensured a uniform cataract surgery and management protocol. Being a retrospective case control study, it also ensured that any special efforts other than specified by the institute were not administered to affect a reduction of infection incidence.

Nationwide surveys and large case series of postcataract endophthalmitis in different countries suggest endophthalmitis incidence from 0.06% to 0.31% [2, 3, 7–23]. Cataract surgery has undergone a significant change in technology and patient care. Briefly, they include ECCE with IOL to phacoemulsification with IOL and ambulatory patient care. This has obviously saved the overall expenses both for the patient and the hospitals, without compromising the surgical outcome and the quality of care [2, 3, 7–10]. 

While the reports of safety and efficacy of the new technology and patient care are available from developed countries, similar reports are sparsely available from less developed countries. This study has documented a higher risk of developing endophthalmitis in the ambulatory care lower socioeconomic group of patients. Important factors associated with this higher incidence may include the residential environment and health education. Poor residential environment and suboptimal health education could have a strong association with higher risk of endophthalmitis.

A major weakness of the study is the study location. A tertiary-care-hospital-based study may not actually reflect the true incidence of postcataract surgery acute endophthalmitis in India, particularly when mass cataract surgery is actively advocated to reduce the back log of cataract blindness. It is also possible that all patients of endophthalmitis, particularly from distant and rural locations, may not have returned for examination. But this possibility is unlikely since most of the noninstitutionalized eye care facilities in India will normally refer these patients to a higher eye care center. We excluded the endophthalmitis patients referred after cataract surgery done outside the institute facilities, so as to obtain uniform pre- and post-operative information. The uniform system adopted in the institute also allowed us to divide the patients into higher and lower socioeconomic groups nearly accurately. We also believe that such a large case control study involving over 46,000 patients and spanning six years probably overcomes some of the deficiencies of the study.

This study suggests that when deciding on to whom to offer ambulatory care cataract surgery and when developing policy related to such surgery, the increased incidence of endophthalmitis in lower socioeconomic class patients compared to those in higher economic categories should be considered. Since a long-term economic benefit lies in one hundred percent ambulatory care, improvement in housing, sanitation, and health education together is likely to improve the surgical outcome.

Cataract is the major cause of reversible blindness [24], and several efforts are made to reduce the cataract blindness [25]. Cataract surgery in itself does not decrease blindness without qualitative effort to improve quality of surgery and postoperative care. Cataract-surgery-related blindness varies from 17% to 43% in India, China and Africa [24, 26–32]. While the efforts of the governmental and Nongovernmental organizations to combat reversible blindness in developing countries are commendable, education on good eye health and care of the operated eye should yield superior outcomes after cataract surgery. An effective and assured ambulatory care will reduce the burden of housing the patients for longer period of time in a hospital. This will also reduce employing health care personnel excess of requirement, thus providing much needed flexibility to the available resources. The modern tools of surgery and management yield better results only when combined with healthy management strategy. In the absence of the later, the technological advancements can never be adequately exploited to advantage in developing countries.

## Figures and Tables

**Table 1:** The final visual acuity by causative organism.

Microorganism	*n* (%)	Final visual acuity				
		20/40	20/50–20/100	<20/200	LP	No. of LP
*S. epidermidis*	16 (43.2)	5	9	1	—	1
*P. aeruginosa*	5 (13.5)	—	—	1	3	1
GPC (other)*	8 (21.6)	1	3	2	1	1
GNB (other)	4 (10.8)	1	1	1	—	1
*P. acnes*	2 (5.5)	—	—	2	—	—
GPB (other)	1 (2.7)	1	—	—	—	—
Fungus	1 (2.7)	—	—	1	—	—

Culture positive	36 (58.1%)	8	13	8	4	4
Culture negative	26 (41.9%)	3	10	10	2	1

*n* = 37 yields in 36 vitreous samples.

GPC: Gram-positive cocci other than *S. epidermidis*.

GNB: Gram-negative bacilli other than *P. aeruginosa.*

GPB: Gram-positive bacilli other than *P. acnes.*

*Two GPC, *α* hemolytic *Streptococcus* and *S. pneumonia, * grew from one sample.

**Table 2:** The incidence of endophthalmitis following cataract surgery.

Patient group		Inpatient	Ambulatory	Total	Fisher exact test
All patients	Total patients	20,039	26,056	46,095	0.054
	Incidence (%)	19 (0.095)	43 (0.165)	62 (0.135)	

Paying patients	Total patients	10,560	13,167	23,727	0.351
	Incidence	10 (0.095)	19 (0.144)	29 (0.122)	

Nonpaying patients	Total patients	9,479	12,889	22,368	0.111
	Incidence	9 (0.095)	24 (0.186)	33 (0.148)	

**Table 3:** Distribution of exposure factors between cases of endophthalmitis and their controls (univariate analysis).

Independent factor	Category	Cases	Controls	Fisher exact test	Unadjusted odds ratio
		*n* = 62	*n* = 310		
Age	—	58 ± 14	59 ± 13	0.600	0.99 (0.77–1.02)

Gender	Female	24; 38.7%	134; 43.2%	0.574	1
	Male	38: 61.3%	176; 56.8%		1.21 (0.69–2.11)

Residence*	Out of city	34; 54.8%	213; 68.7%	0.040	1
	Inside city	28; 45.2%	97; 31.3%		1.81 (1.04–3.15)

Diabetes	No	53; 85.5%	256; 82.6%	0.711	1
	Yes	09; 14.5%	54; 17.4%		0.81 (0.37–1.73)

HTN	No	54; 87.1%	253; 81.6%	0.362	1
	Yes	08; 12.9%	57; 18.4%		0.66 (0.3–1.46)

Care*	Inpatient	19; 30.6%	143; 46.1%	0.025	1
	Ambulatory	43; 69.4%	167; 53.9%		1.94 (1.08–3.48)

Paying status	Paying	28; 45.2%	181; 58.4%	0.068	1
	Nonpaying	34; 54.8%	129; 41.6%		1.7 (0.98–2.95)

Surgery*	ECCE	46; 74.2%	255; 82.3%	0.157	1
	Phaco	16; 25.8%	55; 17.7%		1.61 (0.85–3.06)

Surgeon	Fellow	13; 21.0%	76; 24.5%	0.627	1
	Faculty	49; 79.0%	234; 75.5%		1.22 (0.63–2.38)

*Factors used in multivariate analysis.

**Table 4:** Multivariate analysis of factors associated with postcataract endophthalmitis.

Independent Factor	Category	Coeff. ± SE	*P* value (score statistic)	Odds ratio (95% Cl)
Patient care	Inpatient Ambulatory	−1.16 ± 0.99	0.239	1 0.31 (0.05–2.17)

Paying status	Paying patient Nonpaying patient	−1.41 ± 1.06	0.187	1.000 0.25 (0.03–1.98)

Interaction of patient care and paying status		1.29 ± 0.62	0.038	3.63 (1.07–12.29)

Residence location	Outside city Within city	0.82 ± 0.3	0.007	1.000 2.27 (1.26–4.11)

Constant		−1.84 ± 1.69	0.276	

**Table 5:** Adjusted odds ratio of endophthalmitis with ambulatory patient care in paying and nonpaying patients.

Factor categories Patient care	Paying status	Coeff. ± SE	*P*	Odds ratio (95% CI)
All Inpatients				1

Ambulatory	Paying patients	0.13 ± 0.44	0.769	1.14 (0.48–2.69)
	Nonpaying patients	1.42 ± 0.43	0.001	4.13 (1.77–9.63)

**Table 6:** Adjusted odds ratio of endophthalmitis in nonpaying patients with Inpatient and ambulatory patient care.

Factor categories Paying status	Patient Care	Coeff. ± SE	*P*	Odds ratio (95% CI)
All Inpatients				1

Nonpaying patients	Inpatients	−0.12 ± 0.5	0.815	0.89 (0.34–2.35)
	Ambulatory	1.17 ± 0.36	0.001	3.23 (1.58–6.6)
